# Endoscopic negative pressure therapy as stand-alone treatment for perforated duodenal diverticulum: presentation of two cases

**DOI:** 10.1186/s12876-021-02018-7

**Published:** 2021-11-21

**Authors:** Dörte Wichmann, Kai Tobias Jansen, Flurina Onken, Dietmar Stüker, Emanuel Zerabruck, Christoph R. Werner, Can Yurttas, Karolin Thiel, Alfred Königsrainer, Markus Quante

**Affiliations:** 1 Department of General, Visceral and Transplantation Surgery, University Hospital of Tübingen, Hoppe-Seyler-Str. 3, 72076 Tübingen, Germany; 2 Interdisciplinary Endoscopic Unit, University Hospital of Tübingen, Hoppe-Seyler-Str. 6, 72076 Tübingen, Germany; 3 Department of Internal Medicine I, Gastroenterology, Hepatology, Gastrointestinal Oncology, Infectiology and Geriatrics, University Hospital of Tübingen, Otfried-Müller-Str. 10, 72076 Tübingen, Germany

**Keywords:** Duodenal diverticulum perforation, Endoscopic negative pressure therapy, Endoscopic vacuum therapy, Spontaneous duodenal perforation

## Abstract

**Background:**

Endoscopic negative pressure therapy is a novel and successful treatment method for a variety of gastrointestinal leaks. This therapy mode has been frequently described for rectal and esophageal leakages. Duodenal diverticular perforations are rare but life-threatening events. The early diagnosis of duodenal diverticular perforation is often complicated by inconclusive symptoms. This is the first report about endoscopic negative pressure therapy in patients with perforated duodenal diverticula.

**Case presentation:**

We present two cases of duodenal diverticula perforations treated with endoscopic negative pressure therapy as stand-alone treatment. Start of symptoms varied from one to three days before hospital admission. Early sectional imaging led to the diagnosis of duodenal diverticular perforation. Both patients were treated with endoluminal endoscopic negative pressure therapy with simultaneous feeding option. Three respective changes of the suction device were performed. Both patients were treated with antibiotics and antimycotics during their hospital stay and be discharged from hospital after 20 days.

**Conclusions:**

This is the first description of successful stand-alone treatment by endoscopic negative pressure therapy in two patients with perforated duodenal diverticulum. We thus strongly recommend to attempt interventional therapy with endoluminal endoscopic negative pressure therapy in patients with duodenal diverticular perforations upfront to surgery.

**Supplementary Information:**

The online version contains supplementary material available at 10.1186/s12876-021-02018-7.

## Background

Duodenal diverticula are present in 22% of the population [1]. A very rare but life-threatening complication of duodenal diverticula is possible perforation, which is associated with a mortality rate of up to 20% [2–4]. The clinical symptoms of a duodenal diverticula perforation (DDP) are often non-specific [5–7]. Early CT diagnosis represents the gold standard for detecting the localization of the perforation and further therapy planning [8]. Corresponding radiological findings may include duodenal wall thickening, depiction of the diverticulum, fluid and air collections, and retroperitoneal abscesses. In most cases, spontaneous DDP are localized at juxtapapillary position [7, 9].

Compared with iatrogenic or postoperative duodenal leakage, spontaneous DDP are very rare [3]. According to the hitherto available literature, the majority of patients suffering from DDP is undergoing surgical therapy. Of note, there are only a few case reports about successful conservative therapy of this condition [7, 10–12].

**Fig. 1:**
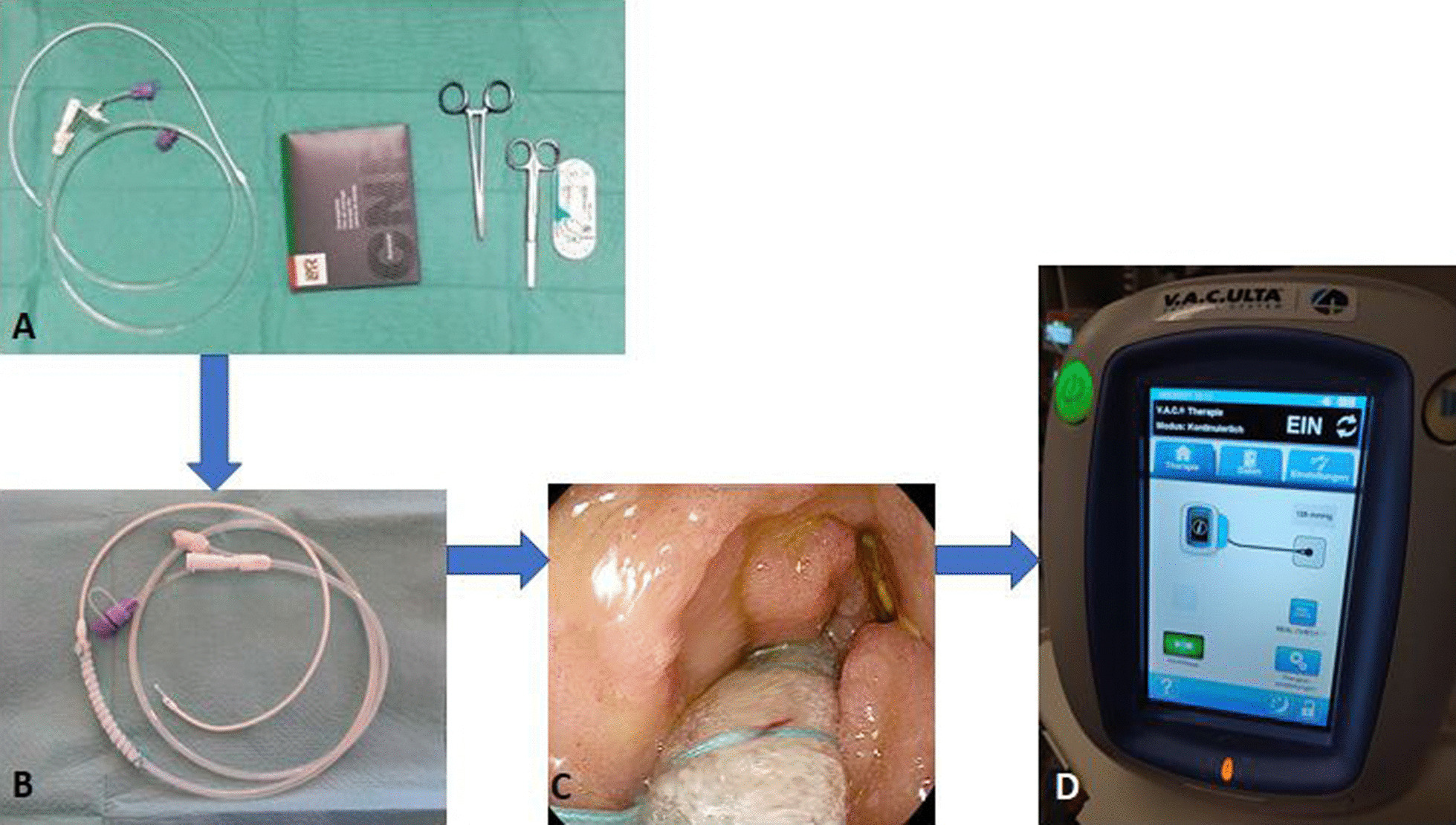
A graphical flowchart of used material for the implementation of the ENPT in the presented patients. **a** All used materials for the preperation of the OFD are shown. FREKA Trelumina (Fresenius Kabi Deutschland GmbH, Germany) naso-jejunal tube. Suprasorb CNP Drainage Folie (Lohmann & Rauscher GmbH & Co. KG, Germany). Suture (Mersilene 1,0; Ethicon Inc. J + J Medical N.V., Belgium). Pin holder and scissors. **b** Prepared tube with wrapped area in position of the gastral perforations. **c** Endoscopic view while the placement of the wrapped tube into the duodenum. **d** Setting of the electric vacuum pump (V.A.C. Ulta; KCI Medical GmbH, Germany)

Endoscopic negative pressure therapy (ENPT) is an effective treatment strategy for various defects of the gastrointestinal tract [13]. The functional principle is based on an open-pore suction device (OPSD), like an open-pore polyurethan sponge, which is placed around a perforated drainage tube, for instance a nasogastric tube. This drainage tube is connected to a vacuum source. An electric vacuum pump with pressure monitoring is recommended for ENPT in the upper GI [14]. In Fig. 1a graphical flow chart of procedures and materials is presented. The OPSD could be placed endoluminal or intracavitary. It is a relatively new therapeutic method, which was introduced in 2000 and rapidly spread due to good clinical results. ENPT improves local perfusion, resolution of interstitial wound edema, removal of fluids, and debridement of the wound base. ENPT is also named EndoVac-therapy or endoscopic vacuum therapy (EVAC or EVT). Different open-pore materials are available for the ENPT. Often, the endoscopist prepares the appropriate OPSD for ENPT from a tube and an open-pored material himself. In the article “Tips and tricks for endoscopic negative pressure therapy” G. Loske introduced different open-pore elements based on the open-pore film (CNP^®^-film = Suprasorb CNP^®^ Drainage Film; Lohmann & Rauscher International GmbH & Co.KG, Rengsdorf, Germany) and polyurethane sponge [14]. The use of feeding tubes for ENPT in endoluminal position can ensure patients’ enteralization for the duration of therapy [15].

Here, we present the first report about successful ENPT as stand-alone treatment for DDP in two patients.

## Case presentation

**Fig. 2:**
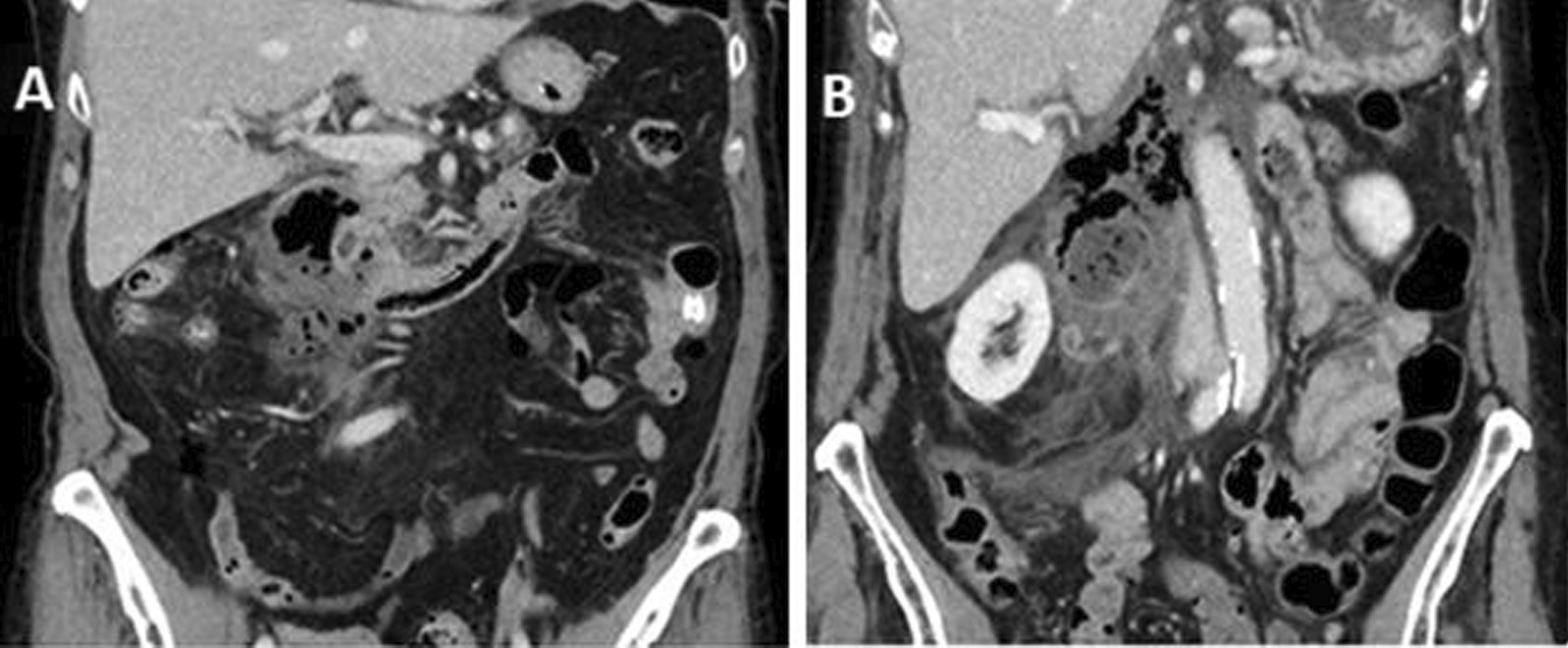
Primary CT-scan of Patient #1 (**a**) and Patient #2 (**b**)

We report about two female patients with spontaneous DDP. Demographic details, medical history and symptoms are listed in Table 1. In Fig. 2, CT-scan at time of hospital admission of both patients is shown. At the first gastroscopy in both patients an acute duodenitis with pus in pars ascendens duodeni was founded. The perforations were not visualized in both cases.

**Table 1:** Patients characteristics

	Patient #1	Patient #2
Age	69	82
Pre-existing conditions	None	None
Delay between start of symptoms and hospital admission	4	1
Laboratory findings	WBC 13,300/µl	WBC 6300/µl
	CRP 30.96 mg/dl	CRP 0.25 mg/dl
	Serum bilirubin 0.6 mg/dl	Serum bilirubin 1.5 mg/dl
Findings in primary sectional imaging	Hollow organ perforation with free retroperitoneal air. Air and fluid retention along the dorsal circumferential pars II duodeni	Perforated duodenal diverticulum with compression of the papillary region

After imaging, both patients underwent initial endoscopic examination by gastroscopies under sedation and were each given a feeding tube with endoluminal OPSD. Reason for first-line endoscopic treatment approach was the desire to avoid laparotomy with opening of retroperitoneum during the acute inflammation. In parallel, antibiotic and antifungal treatment was promptly established.

We created OPSD using 3 ml enteral feeding tubes (Freka Trelumina^®^, Fresenius Kabi Deutschland GmbH, Bad Homburg, Germany) and a cut piece of CNP^®^ film of 2 × 10 cm (CNP^®^-film = Suprasorb CNP^®^ Drainage Film; Lohmann & Rauscher International GmbH & Co.KG, Rengsdorf, Germany). The prepared tube was guide wired placed deep duondenal. The wrapped gastral parts of the tubes were placed into the duodenum. Endoscopic control of the right position was realized.

The gastral tubes were connected to an electric vacuum pump with pressure monitoring (KCI V.A.C.^®^ Ultra Therapy Unit, KCI USA Inc., San Antonio, Texas, United States), according to the description of a fashioned device using a two lumen tube as already described [15]. The predetermined vacuum was 125 mmHg according to the usually used negative pressure for ENPT in the gastrointestinal tract [14].

A diagnostic re-endoscopy was performed once a week. In both patients one additional re-endoscopy for device dislocation was performed. If a persistent perforation was suspected a new OPSD according to the described device was placed into the duodenum. In each case, three respective changes of the system were performed with a total therapy duration of 16 and 17 days. Endoscopic examinations were performed under sedation. Treatment relevant aspects are listed in Table 2.

**Table 2:** Treatment relevant aspects

	Patient #1	Patient #2
Antibiotic Management	5 days cefotaxime, metronidazole and fluconazol	5 days piperacillin and tazobactam and fluconazol
Endoscopic Management	OPSD using 3 ml feeding tube, end of therapy after good progress in CT-imaging	OPSD using 3 ml feeding tube, concluding diverticulography to exclude a persistent perforation
Number of changes of the OPSD	4	4
Number of progress CT	3	3
Length of hospital stay	20 days	20 days
Adverse events	One accidental dislocation of the tube	One accidental dislocation of the tube
Follow-up telephone survey 6 weeks after hospital discharge	Yes, well-being, supplies itself completely independently	Yes, well-being, supplies itself completely independently, occasional vertigo

**Fig. 3:**
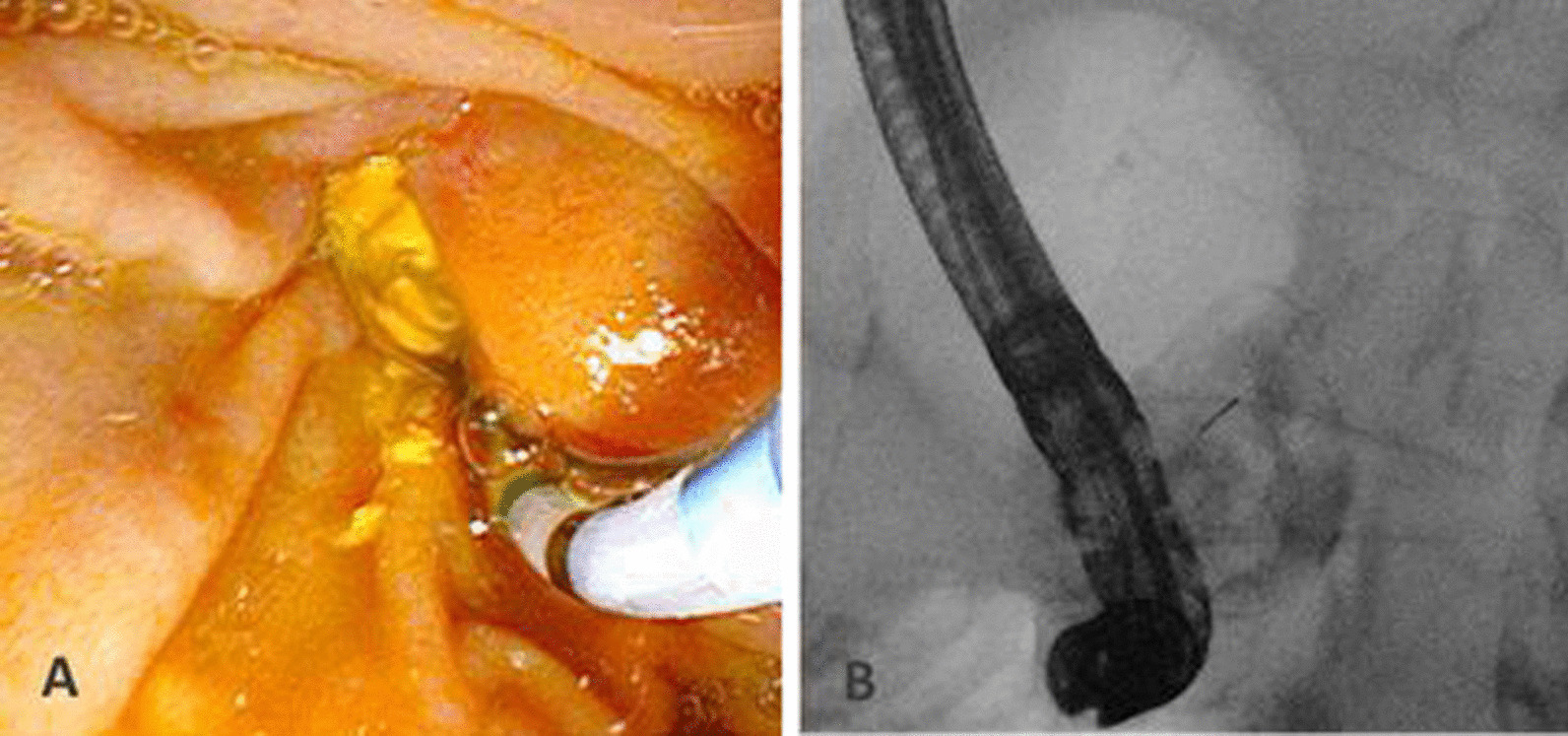
Endoscopic image (**a**) at the time of the diverticulography (**b**)

**Fig. 4:**
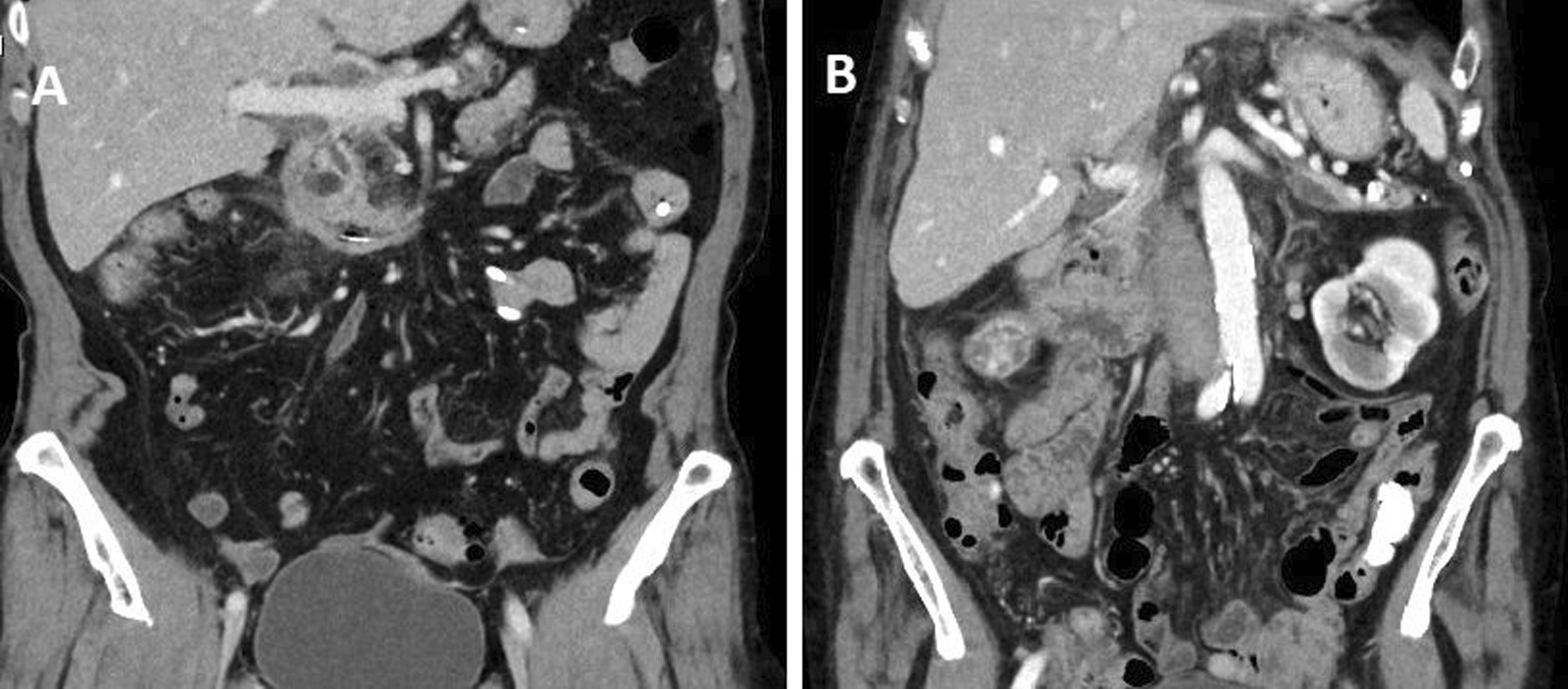
Last CT-scan prior discharge of Patient #1 (**a**) and Patient #2 (**b**)

Antibiotic and antifungal therapy was terminated after five days. In both patients, three follow-up CT scans were performed to monitor the therapy. Fortunately, both patients recovered quickly, were mobile, and were able to drink sips. Patient #2 also underwent a final diverticulography to ensure that the perforation was closed (Fig. 3). After removal of the OPSD both patients were further monitored in the hospital setting for three and four days, respectively. The patients were discharged when food intake and digestion were well established. In Fig. 4 the CT-scan prior discharge is shown in contrast to Fig. 2.

Six weeks after discharge a telephone interview was performed with both patients. They reported on good clinical conditions while still feeling physically weak. No re-admissions to hospital or further episodes of abdominal pain had occurred. Both patients were grateful that they could be treated without surgery.

## Discussion and conclusions

Spontaneous duodenal diverticular perforations are rare but life-threatening events [6]. A literature search for the term “duodenal diverticular perforation” was performed in PubMed. We screened all articles published since 1990 and found a total of 19 case reports and case series describing the treatment of 73 patients (Additional file 1: Table S1). Most common therapy in DDP was surgical in 62 of 73 cases (84.93%). Here, various described operative modii have been reported from open duodenal resection [3] to laparoscopic diverticulectomies [16]. Conservative treatment including nil per ore and antibiotic therapy was described as successful in 11 cases. Overall mortality rate in patients suffering from DDP reported was 10.34%. However, these results may be positively biased since case reports are mainly written in cases that had a positive outcome. There are relevant differences between DDP, and duodenal perforations caused by other etiologies:

1. The absence of a previous trauma or gastrointestinal intervention, which may indicate the presence of perforation in the upper GI-tract. Early diagnosis of a DDP is complicated.
2. The duodenal blood circulation is not affected in cases of DDP compared to anastomotic insufficiencies.
3. The typical localization of the perforation is retroperitoneal starting from pars ascendens duodeni.

ENPT in intracavitary and endoluminal position is increasingly used due to its excellent results in different leakages of the upper and lower gastrointestinal tract [13, 17]. ENPT in duodenal position has been described by Loske, Glatz and Yoo [18–20]. These authors described the use of ENPT in cases of postoperative or iatrogenic leakages of the duodenum.

To the best of our knowledge, we are the first to report on successful ENPT in patients with DDP. Especially for retroperitoneal perforations, this therapy mode allows good drainage of possible abscesses in addition to fluid removal. Of critical importance for the patients, enteralization was ensured during the time of ENPT. Based on the reported results, we strongly recommend to attempt primary endoscopic therapy using ENPT for DDP.

## Supplementary Information

**Additional file 1**. Analysis of existing literature.

## Data Availability

The datasets used and/or analyzed during the current study are available from the corresponding author on reasonable request.

## Abbreviations

CRP: C-reactive protein
DDP: Duodenal diverticular perforation
ENPT: Endoscopic negative pressure therapy
GI: Gastrointestinal
OPSD: Open-pore suction device
WBC: White blood cells
