# Supplementary material for: Endoscopic negative pressure therapy as stand-alone treatment for perforated duodenal diverticulum: presentation of two cases
Source: BMC Gastroenterol. 2021 Nov 21;21:436. doi: 10.1186/s12876-021-02018-7 (PMC8607673; doi:10.1186/s12876-021-02018-7)
Supplement: Supplementary file 1 — Additional file 1. Analysis of existing literature. [file 12876_2021_2018_MOESM1_ESM.docx]

| Year of publishing | First author | Title | Number of included patients | Number of surgical treated patients | Number of conservatively treated patients | Mortality rate |
| --- | --- | --- | --- | --- | --- | --- |
| 2020 | Maki [1] | Repair of a perforated duodenal diverticulum using intraduodenal suture in a 94-year-old woman | 1 | 1 | 0 | 0% |
| 2020 | Myosidis [2] | The challenging diagnosis and treatment of duodenal diverticulum perforation: a report of 2 cases | 2 | 1 | 1 | 0% |
| 2019 | Sahned [3] | Duodenal Diverticulitis: To operate or not to operate? | 1 | 1 | 0 | 0% |
| 2018 | Khan [4] | Duodenal Diverticular Perforation after Small Bowel Obstruction: A case report | 1 | 1 | 0 | 100% |
| 2016 | Yeh [5] | Laparoscopic resection of perforated duodenal diverticulum - a case report and literature reviw | 15 | 14 | 1 | 0% |
| 2014 | Haboubi [6] | Perforated duodenal diverticulae: importance for the surgeon and gastroenterologiest | 1 | 1 | 0 | 0% |
| 2013 | Rossetti [7] | Perforated duodenal diverticulum, a rae complication of a common pathology: a seven patient case series | 7 | 7 | 0 | 14.3% |
| 2008 | Ames [8] | Perforated duodenal diverticulum: clinical and imaging findings in eight patients | 8 | 5 | 3 | 25% |
| 2012 | de Perrot [9] | The complicated duodenal diverticulum: retrospective analysis of 11 cases | 11 | 6 | 5 | 9.1% |
| 2020 | Shimada [10] | Perforated duodenal diverticulum successfully treated with a combination of surgical and endoscopic nasobiliary and nasopancreartic drain: a case report | 1 | 1 | 0 | 0% |
| 2006 | Mathis [11] | Operative management of symptomatic duodenal diverticula | 10 | 10 | 0 | 1 |
| 2015 | Song [12] | Management of Perforated Duodenal Diverticulum: Report of Two Cases. | 2 | 1 | 1 | 0% |
| 2014 | Koh [13] | Perforated duodenal diverticulum as an unusual sequela of intestinal obstruction | 1 | 1 | 0 | 0% |
| 1999 | Tsukamoto [14] | Perforated duodenal diverticulum: report of two cases. | 2 | 1 | 1 | 0% |
| 2006 | Castellvi [15] | Perforated duodenal diverticulum | 1 | 1 | 0 | 0% |
| 1993 | Gugliemli [16] | The perforation of a para-Vater's duodenal diverticulum (a report of 2 clinical cases) | 1 | 1 | 0 | 0% |
| 1990 | Trondsen [17] | Surgical management of duodenal diverticula | 5 | 5 | 0 | 0% |
| 2001 | Gulotta [18] | Perforated duodenal diverticulum: report of a case]. | 1 | 1 | 0 | 0% |
| 2017 | Tamura [19] | Duodenal diverticulitis accompanied by abscess formation treated successfully using an endoscopic nasobiliary drainage catheter: a case report. | 1 | 1 | 0 | 0% |

1. Maki H, Yuasa Y, Matsuo Y, Mori O, Tomibayashi A (2020) Repair of a perforated duodenal diverticulum using intraduodenal suture in 94 year old woman: A case report. Int J Surg Case Rep 71**:**163-167

2. Moysidis M, Paramythiotis D, Karakatsanis A, Amanatidou E, Psoma E, Mavropoulou X, Michalopoulos A (2020) The challenging diagnosis and treatment of duodenal diverticulum perforation: a report of two cases. BMC Gastroenterol 20**:**5

3. Sahned J, Hung Fong S, Mohammed Saeed D, Misra S, Park IS (2019) Duodenal Diverticulitis: To Operate or Not To Operate? Cureus 11**:**e6236

4. Khan K, Saeed S, Maria H, Sbeih M, Iqbal F, Ramcharan A, Donaldson B (2018) Duodenal Diverticular Perforation after Small Bowel Obstruction: A Case Report. Case Rep Surg 2018**:**6197828

5. Yeh TC (2016) Laparoscopic resection of perforated duodenal diverticulum - A case report and literature review. Int J Surg Case Rep 28**:**204-210

6. Haboubi D, Thapar A, Bhan C, Oshowo A (2014) Perforated duodenal diverticulae: importance for the surgeon and gastroenterologist. BMJ Case Rep 2014

7. Rossetti A, Christian BN, Pascal B, Stephane D, Philippe M (2013) Perforated duodenal diverticulum, a rare complication of a common pathology: A seven-patient case series. World J Gastrointest Surg 5**:**47-50

8. Ames JT, Federle MP, Pealer KM (2009) Perforated duodenal diverticulum: clinical and imaging findings in eight patients. Abdom Imaging 34**:**135-139

9. de Perrot T, Poletti PA, Becker CD, Platon A (2012) The complicated duodenal diverticulum: retrospective analysis of 11 cases. Clin Imaging 36**:**287-294

10. Shimada A, Fujita K, Kitago M, Ichisaka S, Ishikawa K, Kikunaga H, Kumai K, Miura H (2020) Perforated duodenal diverticulum successfully treated with a combination of surgical drainage and endoscopic nasobiliary and nasopancreatic drainage: a case report. Surg Case Rep 6**:**129

11. Mathis KL, Farley DR (2007) Operative management of symptomatic duodenal diverticula. Am J Surg 193**:**305-308; discussion 308-309

12. Song S (2015) Management of Perforated Duodenal Diverticulum: Report of Two Cases. Korean J Gastroenterol 66**:**159-163

13. Koh YX, Chok AY, Wong AS (2016) Perforated duodenal diverticulum as an unusual sequelae of intestinal obstruction. ANZ J Surg 86**:**516-517

14. Tsukamoto T, Ohta Y, Hamba H, Sasaki Y, Tokuhara T, Kubo S, Hirohashi K, Kinoshita H (1999) Perforated duodenal diverticulum: report of two cases. Hepatogastroenterology 46**:**1755-1758

15. Castellvi J, Pozuelo O, Vallet J, Sueiras A, Gil V, Espinosa J, Pi F (2006) [Perforated duodenal diverticulum]. Cir Esp 80**:**174-175

16. Guglielmi A, Veraldi GF, Leopardi F, Frameglia M, Boni M (1993) [The perforation of a para-Vater's duodenal diverticulum (a report of 2 clinical cases)]. Ann Ital Chir 64**:**309-312; discussion 313

17. Trondsen E, Rosseland AR, Bakka AO (1990) Surgical management of duodenal diverticula. Acta Chir Scand 156**:**383-386

18. Gulotta G, Agosta G, Romano G (2001) [Perforated duodenal diverticulum: report of a case]. Chir Ital 53**:**255-258

19. Tamura Y, Hayakawa M, Isogawa M, Togashi T, Igarashi M, Takahashi S, Aoyagi Y (2017) Duodenal diverticulitis accompanied by abscess formation treated successfully using an endoscopic nasobiliary drainage catheter: a case report. Clin J Gastroenterol 10**:**240-243
